# The antifungal Aureobasidin A and an analogue are active against the protozoan parasite *Toxoplasma gondii* but do not inhibit sphingolipid biosynthesis

**DOI:** 10.1017/S0031182017000506

**Published:** 2017-05-10

**Authors:** A. Q. I. ALQAISI, A. J. MBEKEANI, M. BASSAS LLORENS, A. P. ELHAMMER, P. W. DENNY

**Affiliations:** 1 Department of Biosciences, Lower Mountjoy, Stockton Road, Durham DH1 3LE, UK; 2 Biology Department, College of Science, University of Baghdad, Baghdad, Iraq; 3 Aureogen Biosciences Inc, 4717 Campus Drive Suite 2300,Kalamazoo, MI 49008, USA

**Keywords:** *Toxoplasma*, sphingolipid biosynthesis, Aureobasidin A, bradyzoite

## Abstract

*Toxoplasma gondii* is an obligate intracellular protozoan parasite of the phylum Apicomplexa, and toxoplasmosis is an important disease of both humans and economically important animals. With a limited array of drugs available there is a need to identify new therapeutic compounds. Aureobasidin A (AbA) is an antifungal that targets the essential inositol phosphorylceramide (IPC, sphingolipid) synthase in pathogenic fungi. This natural cyclic depsipeptide also inhibits *Toxoplasma* proliforation, with the protozoan IPC synthase orthologue proposed as the target. The data presented here show that neither AbA nor an analogue (Compound 20), target the protozoan IPC synthase orthologue or total parasite sphingolipid synthesis. However, further analyses confirm that AbA exhibits significant activity against the proliferative tachyzoite form of *Toxoplasma*, and Compound 20, whilst effective, has reduced efficacy. This difference was more evident on analyses of the direct effect of these compounds against isolated *Toxoplasma*, indicating that AbA is rapidly microbicidal. Importantly, the possibility of targeting the encysted, bradyzoite, form of the parasite with AbA and Compound 20 was demonstrated, indicating that this class of compounds may provide the basis for the first effective treatment for chronic toxoplasmosis.

## INTRODUCTION

**Fig. 1.**
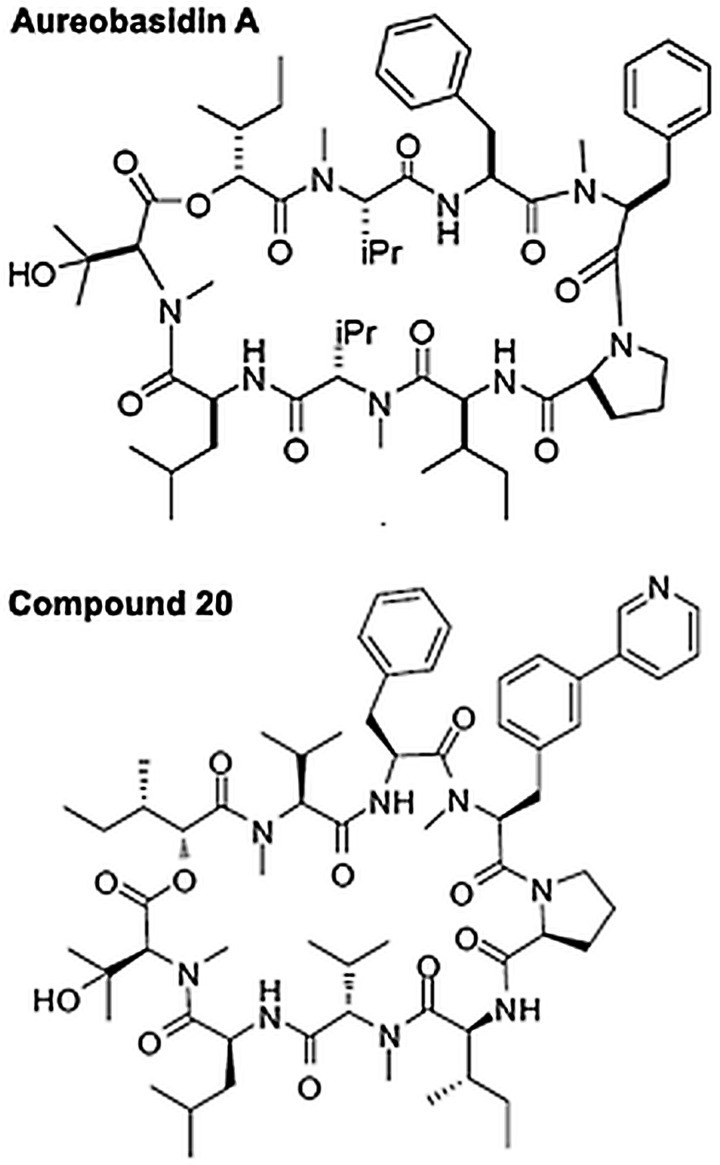
The structures of the cyclic depsipeptide compounds Aureobasidin A and its analogue Compound 20 (Wuts *et al.* 2015).

Aureobasidin A (AbA; Fig. 1) is a cyclic depsipeptide antifungal antibiotic isolated from the fungus *Aureobasidium pullulans* R106 (Ikai *et al*. 1991; Takesako *et al*. 1991). Resistance in *Saccharomycin cerevisiae* was found to be conferred by dominant negative mutations in the Aureobasidin resistance (AUR1) gene (Heidler and Radding, 1995). Subsequently, AUR1 was identified as encoding the essential inositol phosphorylceramide (IPC) synthase activity in fungi (Nagiec *et al*. 1997). AbA has been shown to be an irreversible inhibitor of the *S. cerevisiae* IPC synthase, acting in a time dependant manner (Aeed *et al*. 2009), with the toxic effects associated with both a build up of the bioactive substrate ceramide and the deprivation of IPC (Cerantola *et al*. 2009). Recent efforts have utilized a semi-synthetic approach to generate analogues of AbA which demonstrate improved activity against some pathogenic fungal species, for example *Aspirgillus fumigatus* (Wuts *et al*. 2015).

IPC is an essential sphingolipid found in fungi, plants and some protozoa (Young *et al*. 2012). In contrast, mammals lack IPC and instead synthesize sphingomyelin (SM) as their major sphingolipid species using SM synthase (Huitema *et al*. 2004). Complex sphingolipids, such as IPC and SM, are major components of the outer leaflet of eukaryotic plasma membranes that are thought to be involved, together with sterols, in the formation of micro-domains known as lipid rafts. These rafts have been proposed to function in a diverse array of processes from the polarised trafficking of lipid-modified proteins, to the assembly and activation of signal transduction complexes (Simons and Ikonen, 1997). The non-mammalian nature of IPC synthase makes it an attractive drug target, and it has been validated as such in both the pathogenic fungi and the kinetoplastid protozoa (Georgopapadakou, 2000; Hanada, 2005; Mina *et al*. 2009, 2010).

*Toxoplasma gondii* is an obligate, intracellular protozoan parasite, able to invade and colonize a wide variety of nucleated vertebrate cells. It is a member of the Apicomplexa, a diverse phylum including important pathogens of domestic animals and humans such as *Eimeria* (the etiological agent of coccidiosis in poultry), *Theileria* (East Coast Fever in Cattle), *Cryptosporidium* (diarrhoea) and *Plasmodium* (malaria). In common with other apicomplexans *Toxoplasma* has a complex lifecycle, involving a definitive, feline, host; and both rapidly proliferative, tachyzoite forms (all tissues in acute disease) and slowly dividing, bradyzoite forms (muscle and brain tissue cysts in chronic disease) (Dubey, 1977). *Toxoplasma* is an opportunistic pathogen and is a significant cause of disease (toxoplasmosis) in the immunocompromised: particularly organ transplant recipients, those receiving anti-cancer chemotherapy and AIDS patients (Chowdhury, 1986). *In utero* toxoplasmosis is also a significant cause of congenital defects in humans (Chowdhury, 1986) and spontaneous abortion in economically important domestic animals (Dubey, 1977). The diseases listed above are associated with rapidly dividing, tachyzoite *Toxoplasma,* either directly acquired or the result of the reactivation of a chronic infection. However, in addition, bradyzoite, chronic, toxoplasmosis has been associated with psychiatric disorders, including schizophrenia (Webster *et al*. 2013). The drugs available for acute toxoplasmosis (tachyzoite stage) have various problems with efficacy and safety, furthermore no treatments are available for chronic disease (encysted bradyzoite stage) therefore new therapies are urgently required (Antczak *et al*. 2016).

The synthesis of IPC by *Toxoplasma* was first reported on the basis of metabolic labelling experiments (Sonda *et al*. 2005) and subsequently confirmed using directed mass spectrometry (Pratt *et al*. 2013). In addition, inhibition of parasite IPC synthesis by AbA was indicated and the tractability of this natural compound as a new lead proposed (Sonda *et al.* 2005; Coppens, 2013). Utilising AbA and the availability of a well characterized orthologue with improved pharmacokinetic properties, Compound 20 (Fig. 1, modified with a pyridyl group at AbA position 4; Wuts *et al*. 2015), here we examine the effect of these compounds on the *Toxoplasma* AUR1 orthologue (*Tg*SLS; (Pratt *et al*. 2013) and total sphingolipid biosynthesis; and on the proliferation of both tachyzoite and bradyzoite form parasites. The results demonstrate that whilst both compounds inhibit the proliferation of *Toxoplasma*, neither inhibits *Tg*SLS nor total sphingolipid biosynthesis as previously proposed (Sonda *et al.* 2005; Coppens, 2013). However, despite uncertainty regarding the mode of action, the ability of this class of cyclic depsipeptides to clear encysted bradyzoite-like form *Toxoplasma* from infected tissue culture cells marks them as a possibly unique therapy for chronic toxoplasmosis.

## MATERIALS AND METHODS

### Cell culture

*Toxoplasma gondii* (strains RH-TATi-1 (Meissner *et al*. 2002), RH-HX-KO-YFP2-DHFR (Gubbels *et al*. 2003) and Pru-GRA2-GFP-DHFR (Kim *et al*. 2007) were maintained in Vero, Human Foreskin Fibroblast (HFF) or Chinese Hamster Ovary (CHO) cells grown in DMEM (Invitrogen) supplemented with 10% fetal bovine serum (FBS) at 37 °C and 5% CO_2_. Type II *Toxoplasma* (Pru strain) tachyzoites were differentiated to the bradyzoite-like form in HFF cells via an alkaline shift to pH8 as previously described (Soete *et al*. 1994).

### Metabolic labelling

*Saccharomoyces cerevisiae* and Vero cells were labelled with 5 *µ*m of NBD C_6_-ceramide complexed with Bovine Serum Albumin (BSA) (Invitrogen) for use as controls as previously (Denny *et al*. 2006). *Toxoplasma* tachyzoites were separated from host cell material by filtration through 3 and 5 mm polycarbonate filters (Millipore) after disruption by passage through a narrow gauge needle. Released parasites were then isolated by centrifugation at 1430 ***g*** for 15 min at room temperature, washed and resuspended in serum-free DMEM (Invitrogen) at 10^7^ mL^−1^ and incubated for 1 h at 37 °C before the addition of NBD C_6_-ceramide complexed with BSA to 5 *µ*m, and a further 1 h at 37 °C. For the inhibitor studies, AbA or Compound 20 were added to isolated *Toxoplasma* at 10 *µ*g mL^−1^ and incubated at 37 °C for 1, 4 or 7 h, before the addition of NBD C_6_-ceramide complexed with BSA to 5 *µ*m and a further incubation at 37 °C for 1 h. Lipids were extracted and analysed by HPTLC as previously described (Mina *et al*. 2009).

### Toxoplasma susceptibility assay

HFF cells were seeded at 10^4^ cells per well in 96 well microtitre plates (Nunc). After 18 h at 37 °C isolated *Toxoplasma* RH-HX-KO-YFP2-DHFR (Gubbels *et al*. 2003) were inoculated into the host cells at 6250 parasites per well. Following a further 20 h incubation compounds were added at the appropriate concentrations. In an additional experiment, isolated tachyzoite parasites were pre-treated with compounds for 2 and 8 h, then washed, prior to infection of HFF cells. For bradyzoite assays, the *Toxoplasma* Pru-GRA2-GFP-DHFR (Kim *et al*. 2007) tachyzoites were added at the same concentration but then transformed as described (Soete *et al*. 1994) before the addition of the compounds. Plates were washed after 2 or 8 h, or not, as described in text. The plates were read using a Biotek Synergy H4 plate reader (Ex 510 nm; Em 540 nm) after 6 or 3 days, respectively.

### Yeast susceptibility assay

YPH499-HIS-GAL-AUR1 (a yeast strain in which expression of the essential IPC synthase, AUR1p, is under the control of a galactose promoter) complemented with *Tg*SLS or AUR1 (Denny *et al*. 2006; Pratt *et al*. 2013) were assayed for susceptibility to AbA and Compound 20. The transgenic yeast strains were maintained on SD -HIS -URA agar (0·17% Bacto yeast nitrogen base, 0·5% ammonium sulphate, 2% glucose, containing the appropriate nutritional supplements) at 30 °C. To analyse susceptibility to AbA and Compound 20 plates containing 5 or 10 *µ*g mL^−1^ of the compound were prepared and 10 *µ*L of an aqueous suspension of yeast spotted onto the surface before incubation at 30 °C.

### Transcript analyses

For the mRNA analyses, total RNA from equivalent numbers of CHO cells infected for 72 h with *Toxoplasma* RH-TATi parasites, or non-infected, was extracted using the RNeasy kit (Qiagen) according to the manufacturer's protocol. Following DNase treatment (RQ1, Promega) cDNA was synthesized using the ImProm-II Reverse Transcription System (Promega) according to manufacturer's protocol. Quantitative PCR (qPCR) was performed in a RotorGene^®^ RG3000 (Corbett Research) using SYBR Green Jump-Start Taq Ready Mix (Sigma Aldrich) according to the manufacturer's instructions. The hamster, *Cricetulus griseus, CgLcb2* (encoding subunit 2 of SPT) was amplified using the primer pair – 5′CAGACAACTTTGTTTTCGG3′ and 5′GGGTGGCATTGTAGGGC3′. The reference gene, *CgβTub*, was amplified using the primer pair – 5′TAAAACGACGGCCAG*TG*AGC3′ and 5′TCTCC*TG*GCGAG*TG*C*TG*C3′. The qPCR was carried out in triplicate on 3 replicates with an annealing temperature 55°C for *CgLcb2* and *CgβTub*.

## RESULTS

### Comparing the effect of AbA and its analogue Compound 20 on the proliferation of the Toxoplasma tachyzoite form

**Fig. 2.**
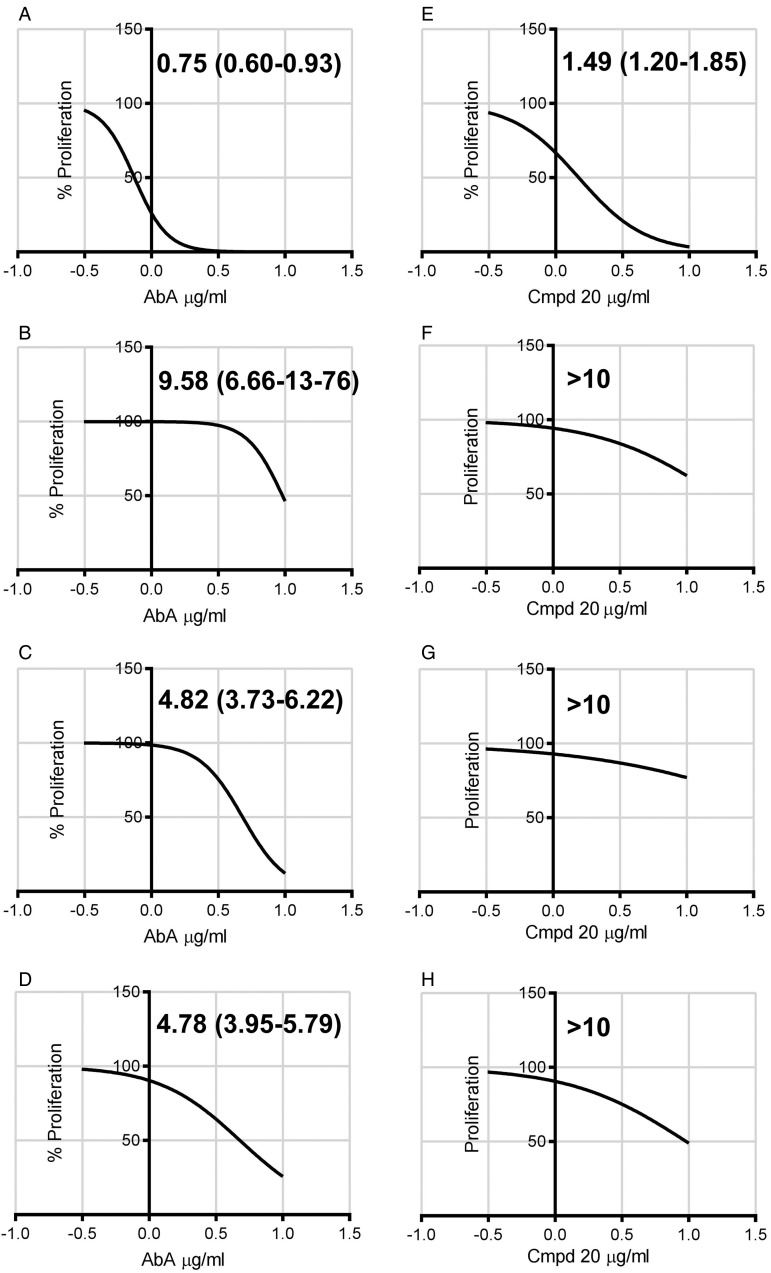
ED_50_ of Aureobasidin A (AbA, A-D) or Compound 20 (Cpmd 20, E-H) – *μ*g mL^−1^; (95% Confidence Interval) – against the Toxoplasma RH tachyzoite form in HFF cells. 6 days post addition of the compounds. In agreement with Sonda *et al.* (2005), both compounds were non-toxic to HHF cells under the conditions employed. A and B: no wash out post-compound addition; C and D: wash out 2 h post-compound addition; E and F: wash out 8 h post-compound addition; G and H: 2 h pre-treatment of isolated parasites pre-infection. Calculated using GraphPad Prism 7, log(inhibitor) *vs* normalized response – Variable slope. >10 *µ*g mL^−1^ – ED_50_ could not be determined. Representative in triplicate dataset.

AbA has previously been shown to inhibit the proliferation of the rapidly dividing, tachyzoite form of *Toxoplasma.* The effective concentration of compound reducing proliferation by 50% (ED_50_) was calculated as 0·3 *µ*g mL^−1^ by cell counting 48 h post infection and 46 h post addition of AbA (Sonda *et al*. 2005). In order to gain a more rapid and robust dataset to facilitate comparative analyses of the efficacy of AbA and Compound 20 we utilised the availability of the yellow fluorescent protein labelled *Toxoplasma*, RH-strain (Gubbels *et al*. 2003). Gubbels *et al*. demonstrated the tractability of this system by comparison with *β*-galactosidase producing parasites. Following validation and parameter setting (data not shown), HFF cells were plated onto 96-well plates and infected with 6250 *Toxoplasma* per well as described in the section Materials and Methods. After 20 h the compounds were added and, without washing, the plate incubated for 144 h (6 days) before fluorescent readings were taken. Following data analyses the ED_50_ was calculated as described (Fig. 2). As can been seen both AbA and Compound 20 showed activity against *Toxoplasma* RH tachyzoites. However, the parent compound (ED_50_ of 0·75, 95% CI 0·60 to 0·93 *µ*g mL^−1^) was slightly more efficacious than its derivative (ED_50_ of 1·49, 95% CI 1·20 to 1·85 *µ*g mL^−1^). This differential activity was even more evident on further analyses. Previously, using an indirect assay (vacuole formation), it has been indicated that the efficacy of AbA against *Toxoplasma* is partially reversible after 24, but not 48 h, exposure (Sonda *et al*. 2005). To further analyse the reversibility of the efficacy of cyclic depsipeptides, the infected HFF cells were washed following 2 and 8 h of compound treatment and proliferation then followed for 6 days as previously (Fig. 2). In keeping with Sonda *et al.* (2005) efficacy was partially reversible, but *Toxoplasma* were clearly susceptible to AbA in an 8 h treatment (ED_50_ of 4·82, 95% CI 3·73 to 6·22 *µ*g mL^−1^), and even 2 h exposure demonstrated some activity (ED_50_ of 9·58, 95% CI 6·66 to 13·76 *µ*g mL^−1^). However, in contrast, the activity of Compound 20 was demonstrated to be almost completely reversible under the conditions employed.

Interestingly, the unrelated kinetoplastid protozoa, *Trypanosoma cruzi* (the causative agent of American Trypanosomiasis or Chagas disease) has also been shown to be sensitive to AbA, with the IPC synthase again proposed as the target (Salto *et al*. 2003). However, enzyme analyses did not confirm this and it was suggested that the compound acts on the host to promote clearance of the parasite (Figueiredo *et al*. 2005). In order to test this hypothesis in *Toxoplasma* infection, tachyzoite parasites were isolated from infected cells as described in the section Materials and Methods and then treated with various concentrations of AbA and Compound 20 prior to washing and infecting host HFF cells. A 2 h treatment again demonstrated that AbA was effective (ED_50_ of 4·78, 95% CI 3·95 to 5·79 *µ*g mL^−1^), whilst the analogue was inactive (Fig. 2). Longer periods post-isolation (8 h) lead to untreated parasites losing infectivity.

### The sensitivity of the Toxoplasma gondii sphingolipid synthase and sphingolipid synthesis per se to AbA and its analogue Compound 20

**Fig. 3.**
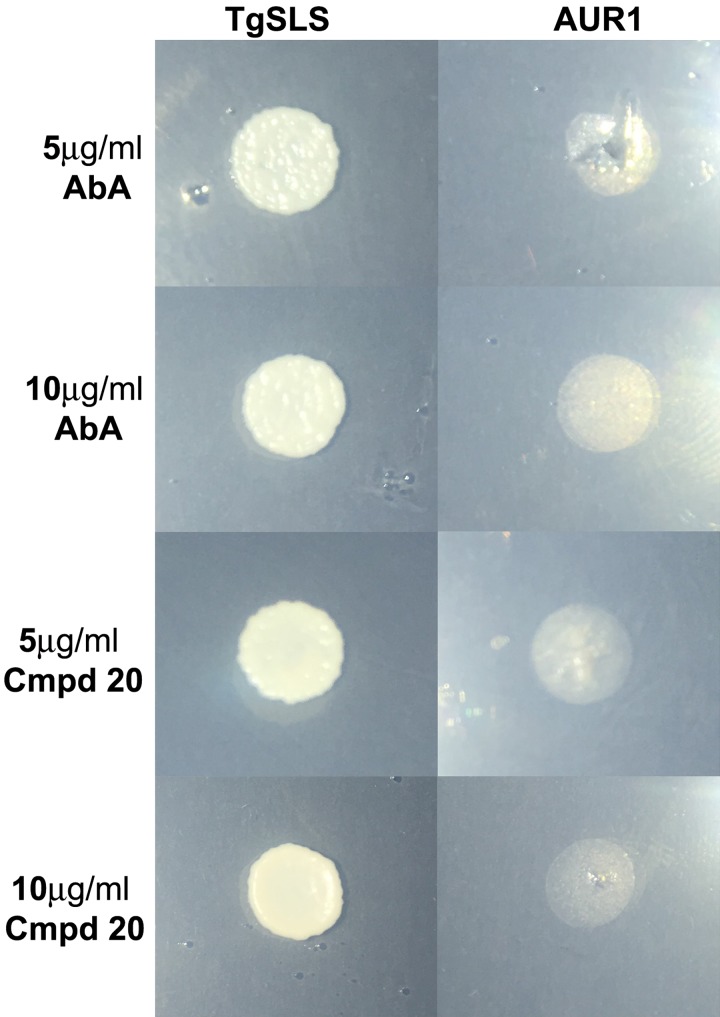
Yeast dependent on the expression of the Toxoplasma AUR1p orthologue *Tg*SLS (YPH499-HIS-GAL-AUR1 pRS426 *Tg*SLS) are resistant to Aureobasidin A (AbA) and Compound 20 (Cmpd 20) at 5 and 10 *µ*g mL^−1^. This contrasts to the sensitivity of yeast dependent on AUR1 expression (YPH499-HIS-GAL-AUR1 pRS426 AUR1).

**Fig. 4.**
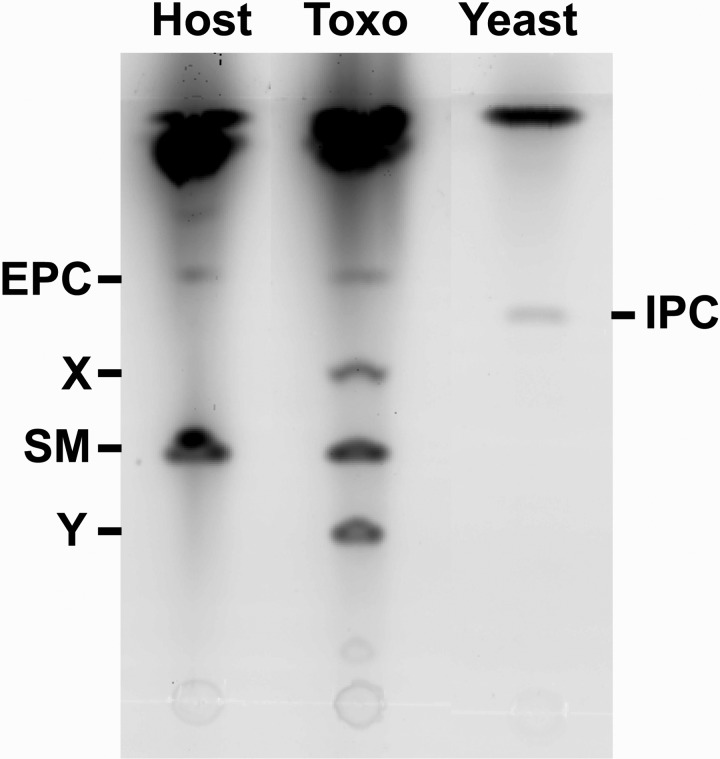
Vero cells (Host), isolated *Toxoplasma* tachyzoites (Toxo) and *Saccharomyces cerevisiae* (Yeast), labelled for 1 h with NBD-C6-ceramide and complex sphingolipids then fractionated by HPTLC. Like the host cells, Toxoplasma parasites synthesize sphingomyelin (SM) and ethanolamine phosphorylceramide (EPC), two unique sphingolipids are also produced (X and Y). However, unlike in *S. cerevisiae*, no labelled inositol phosphorylceramide (IPC) is evident from either host or *Toxoplasma* cells. Representative dataset.

**Fig. 5.**
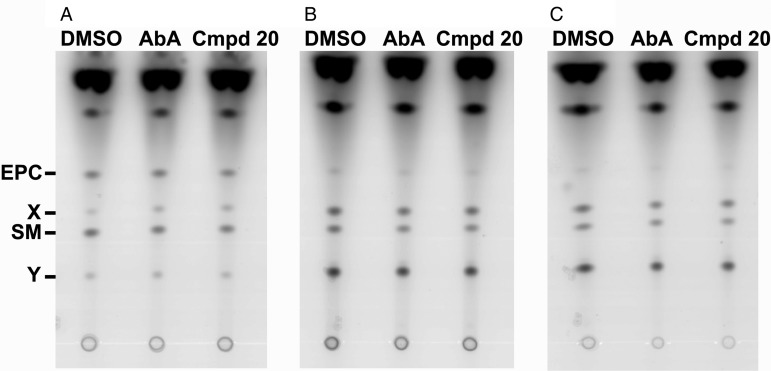
Isolated *Toxoplasma* tachyzoites treated with Aureobasidin A (AbA) and Compound 20 (Cmpd 20) at 10 *µ*g mL^−1^ for 1 (A), 4 (B) and 7 (C) hours before labelling with NBD-C6-ceramide for 1 h. Neither compound affected the complex sphingolipid profile synthesized at any time point when compared with the vehicle control (DMSO). SM – Sphingomyelin (SM); EPC – Ethanolamine PhosphorylCeramide; X and Y – Unclassified sphingolipids. Representative dataset.

Host sphingolipid biosynthesis is unaffected by (Fig. S1) and non-essential for (Pratt *et al*. 2013; Romano *et al*. 2013), *Toxoplasma* proliferation. Therefore, *de novo* synthesis of sphingolipids is an attractive target for new antiprotozoal drug leads. The antifungal sphingolipid (IPC) synthase inhibitor AbA has been proposed to inhibit the *Toxoplasma* orthologue (Sonda *et al.* 2005; Coppens, 2013). However, analyses of an enzyme isolated from *Toxoplasma* demonstrating IPC synthase activity (*Tg*SLS) did not support this conclusion (Pratt *et al*. 2013). Utilizing the previously constructed, auxotropic, *Tg*SLS complemented yeast strains (YPH499-HIS-GAL-AUR1 pRS426 *Tg*SLS, with YPH499-HIS-GAL-AUR1 pRS426 AUR1 as a control), the sensitivity of the protozoan enzyme to AbA and Compound 20 was analysed (Fig. 3). The results clearly demonstrated that the *Toxoplasma* enzyme conferred resistance to yeast against both cyclic depsipeptides at concentrations lethal to yeast reliant on AUR1 activity (5 and 10 *µ*g mL^−1^). However, whilst *Tg*SLS clearly functions as an IPC synthase in yeast and *in vitro, Toxoplasma* have also been demonstrated, by the incorporation of tritiated serine, to synthesize sphingomyelin (SM) and glycosphingolipids (GSLs) (Gerold and Schwarz, 2001). The presence of SM and GSLs in isolated *Toxoplasma* was subsequently confirmed using mass spectrometry (Welti *et al.* 2007; Pratt *et al*. 2013). In addition, relatively high levels of ethanolamine phosphorylceramide (EPC), a non-abundant species in mammalian cells, were also reported (Welti *et al.* 2007; Pratt *et al*. 2013). In light of this synthetic complexity, and the potential of enzymatic diversity, the effect of AbA and Compound 20 on total sphingolipid biosynthesis in *Toxoplasma* was investigated. Labelling isolated *Toxoplasma* with NBD-C_6−_ceramide as described in the section Materials and Methods demonstrated that the parasite synthesized a complex of sphingolipid species, including SM and EPC (co-migrating with mammalian equivalents; Vacaru *et al*. 2013). However, IPC was not evident and 2 other species (X and Y) remain unassigned (Fig. 4). The addition of AbA and Compound 20 at 10 *µ*g mL^−1^ for 1, 4 and 7 h, before 1 h NBD-C_6−_ceramide labelling, had no effect on the synthesis of the sphingolipids compared with controls (Fig. 5). This demonstrated that this class of cyclic depsipeptides do not exert their activity through inhibition or dysregulation of sphingolipid biosynthesis. However, it is notable that the complex sphingolipid profile produced does change as the time post parasite isolation increases, with the levels of labelled lipids X and Y increased at 4 and 7 h, EPC levels decreased and SM levels unchanged (Fig. 5). This indicated that the stress of isolation from the host cell leads to the modification sphingolipid biosynthesis or to catabolism.

### Comparing the efficacy of AbA and its analogue Compound 20 against the encysted Toxoplasma bradyzoite form

**Fig. 6.**
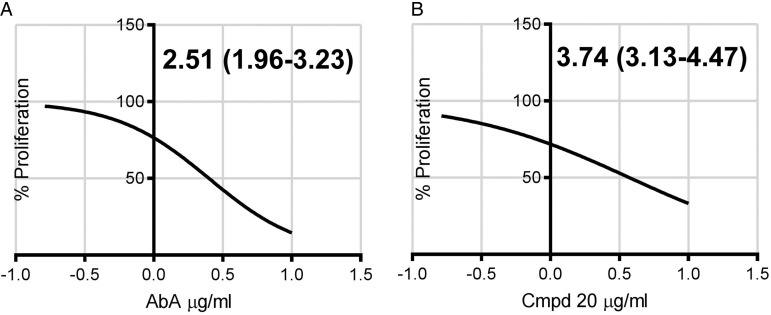
ED_50_ of Aureobasidin A (A, AbA) or Compound 20 (B, Cpmd 20) – *μ*g mL^−1^ (95% Confidence Interval) – against the Toxoplasma Pru bradyzoite form in Human Foreskin Fibroblast (HFF) cells. Three days post addition of the compounds. In agreement with Sonda *et al.* (2005), both compounds were non-toxic to HHF cells under the conditions employed. Calculated using GraphPad Prism 7, log(inhibitor) *vs* normalized response – Variable slope. Representative in triplicate dataset.

With a complete lack of treatments available for chronic disease, in which *Toxoplasma* has reached the encysted bradyzoite stage, new therapies are urgently needed (Antczak *et al*. 2016). Therefore, although the mode of action of the cyclic depsipeptides remains unclear, the efficacy of these compounds against the encysted form of the parasite was analysed. Utilizing the Type II Pru strain of *Toxoplasma* modified to express GFP (Kim *et al*. 2007) we analysed the efficacy of AbA and Compound 20 against HFF cells infected with parasites transformed into a bradyzoite-like stage using an established protocol (Soete *et al*. 1994). Following 3 days of exposure, both compounds demonstrated promising activity against the encysted *Toxoplasma* (Fig. 6), again AbA demonstrated slightly higher efficacy (ED_50_ of 2·51, 95% CI 1·96 to 3·23 *µ*g mL^−1^) than Compound 20 (ED_50_ of 3·74, 95% CI 3·13 to 4·47 *µ*g mL^−1^). This showed that the cyclic depsipeptides may represent promising candidates for therapies to treat both acute and chronic toxoplasmosis.

## DISCUSSION

*Toxoplasma* is an important cause of disease in humans and domestic animals. Whilst there are several drugs available to treat acute (tachyzoite stage) toxoplasmosis, there is a complete absence of effective therapies for chronic disease (encysted bradyzoite stage; Antczak *et al*. 2016). It has been demonstrated that *Toxoplasma* remain able to replicate in CHO cells where the activity of the first and rate limiting step in sphingolipid biosythesis, serine palmitoyltransferase (SPT), was greatly reduced and complex sphinglipid levels consequently lowered (Hanada *et al*. 1992; Pratt *et al*. 2013). In addition, in this study we showed that key enzymes in host (CHO) sphingolipid biosynthesis are unaffected by *Toxoplasma* infection (Fig. S1). Together, these data indicated that targeting the *de novo Toxoplasma* sphingolipid biosynthetic pathway could represent a viable strategy towards the identification of new antiprotozoals. A strategy that could also be applicable to other apicomplexan parasites such as *Plasmodium* spp. (Lauer *et al*. 1995), and one that has is already being investigated for kinetoplastid protozoan pathogens (Denny *et al*. 2006; Mina *et al*. 2009, 2010, 2011).

To these ends it has been suggested that the antifungal cyclic depsipeptide, AbA exerts its effect on *Toxoplasma* by inhibiting a sphinglipid (IPC) synthase, an orthologue of its validated target in yeast (Nagiec *et al*. 1997; Sonda *et al*. 2005). Given the status of the fungal and kinetoplastid IPC synthases as promising drug targets (Young *et al*. 2012), the identification of the *Toxoplasma* orthologue (Pratt *et al*. 2013) led to its consideration as a target for anti-apicomplexan drugs. *Tg*SLS functions as an IPC synthase and the product was identified in parasite extracts using directed mass spectrometry. However, AbA was demonstrated to be non-active against the enzyme activity *in vitro* (Pratt *et al*. 2013).

To investigate this compound class further, here we utilized the availability of AbA and a synthetically modified analogue, Compound 20 (Wuts *et al*. 2015), to test the efficacy and mode of action of these cyclic depsipeptides against *Toxoplasma*. As expected, neither compound inhibited the growth of transgenic yeast dependent on the expression of *Tg*SLS (Fig. 3). Furthermore, the compounds also exhibited no effect on the synthesis of complex sphingolipids in *Toxoplasma* (Fig. 5). Interestingly, no IPC synthesis was apparent indicating that this activity may be low, in tachyzoites at least. However, both SM and EPC (Azzouz *et al*. 2002; Welti *et al*. 2007) were clearly produced, as well as 2 uncharacterised complex sphingolipids (Fig. 4). However, despite this lack of dysregulation of sphingolipid biosythesis, both AbA and Compound 20 are active against the tachyzoite form of the parasite in infected HHF cells. AbA exhibited greater efficacy and, unlike Compound 20, demonstrated a rapid and direct ‘cidal activity against the *Toxoplasma* parasite (Fig. 2). Furthermore, and importantly, both AbA and Compound 20 clear encysted bradyzoite-like form *Toxoplasma* from infected tissue culture at low concentrations (Fig. 6). Given the well established lack of toxicity of these compounds to mammalian cells, coupled with the promising pharmacokinetic properties of Compound 20 (Wuts *et al*. 2015), this class of cyclic depsipeptides may form the basis of a unique therapy for chronic toxoplasmosis and perhaps, some psychiatric disorders.

## Supplementary material

For supplementary material accompanying this paper visit http://dx.doi.org/10.1017/S0031182017000506. click here to view supplementary material

## References

[ref1] Aeed P. A., Young C. L., Nagiec M. M. and Elhammer A. P. (2009). Inhibition of inositol phosphorylceramide synthase by the cyclic peptide Aureobasidin A. Antimicrobial Agents and Chemotherapy 53, 496–504. 19047657 10.1128/AAC.00633-08 PMC2630602

[ref2] Antczak M., Dzitko K. and Dlugonska H. (2016). Human toxoplasmosis-Searching for novel chemotherapeutics. Biomedicine and Pharmacotherapy 82, 677–684. 27470411 10.1016/j.biopha.2016.05.041

[ref3] Azzouz N., Rauscher B., Gerold P., Cesbron-Delauw M. F., Dubremetz J. F. and Schwarz R. T. (2002). Evidence for *de novo* sphingolipid biosynthesis in *Toxoplasma gondii*. International Journal for Parasitology 32, 677–684. 12062486 10.1016/s0020-7519(02)00009-7

[ref4] Cerantola V., Guillas I., Roubaty C., Vionnet C., Uldry D., Knudsen J. and Conzelmann A. (2009). Aureobasidin A arrests growth of yeast cells through both ceramide intoxication and deprivation of essential inositolphosphorylceramides. Molecular Microbiology 71, 1523–1537. 19210614 10.1111/j.1365-2958.2009.06628.x

[ref5] Chowdhury M. N. (1986). Toxoplasmosis: a review. Journal of Medicine 17, 373–396. 3295094

[ref6] Coppens I. (2013). Targeting lipid biosynthesis and salvage in apicomplexan parasites for improved chemotherapies. Nature Reviews Microbiology 11, 823–835. 24162026 10.1038/nrmicro3139

[ref7] Denny P. W., Shams-Eldin H., Price H. P., Smith D. F. and Schwarz R. T. (2006). The protozoan inositol phosphorylceramide synthase: a novel drug target which defines a new class of sphingolipid synthase. Journal of Biological Chemistry 281, 28200–28209. 16861742 10.1074/jbc.M600796200 PMC1817671

[ref8] Dubey J. P. (1977). *Toxoplasma, Hammondia, Besnotia, Sarcocystis*, and other cyst-forming coccidia of man and animals In Parasitic Protozoa (ed. Kreier J. P.), pp. 101–237. Academic Press, New York.

[ref9] Figueiredo J. M., Dias W. B., Mendonca-Previato L., Previato J. O. and Heise N. (2005). Characterization of the inositol phosphorylceramide synthase activity from *Trypanosoma cruzi*. Biochemical Journal 387, 519–529. 15569002 10.1042/BJ20041842 PMC1134981

[ref10] Georgopapadakou N. H. (2000). Antifungals targeted to sphingolipid synthesis: focus on inositol phosphorylceramide synthase. Expert Opinions on Investigative Drugs 9, 1787–1796. 10.1517/13543784.9.8.1787 11060777

[ref11] Gerold P. and Schwarz R. T. (2001). Biosynthesis of glycosphingolipids de-novo by the human malaria parasite *Plasmodium falciparum*. Molecular and Biochemical Parasitology 112, 29–37. 11166384 10.1016/s0166-6851(00)00336-4

[ref12] Gubbels M. J., Li C. and Striepen B. (2003). High-throughput growth assay for *Toxoplasma gondii* using yellow fluorescent protein. Antimicrobial Agents and Chemotherapy 47, 309–316. 12499207 10.1128/AAC.47.1.309-316.2003 PMC149035

[ref13] Hanada K. (2005). Sphingolipids in infectious diseases. Japanese Journal of Infectious Diseases 58, 131–148. 15973004

[ref14] Hanada K., Nishijima M., Kiso M., Hasegawa A., Fujita S., Ogawa T. and Akamatsu Y. (1992). Sphingolipids are essential for the growth of Chinese hamster ovary cells. Restoration of the growth of a mutant defective in sphingoid base biosynthesis by exogenous sphingolipids. Journal of Biological Chemistry 267, 23527–23533. 1429697

[ref15] Heidler S. A. and Radding J. A. (1995). The AUR1 gene in *Saccharomyces cerevisiae* encodes dominant resistance to the antifungal agent Aureobasidin A (LY295337). Antimicrobial Agents and Chemotherapy 39, 2765–2769. 8593016 10.1128/aac.39.12.2765 PMC163026

[ref16] Huitema K., Van Den Dikkenberg J., Brouwers J. F. and Holthuis J. C. (2004). Identification of a family of animal sphingomyelin synthases. EMBO Journal 23, 33–44. 14685263 10.1038/sj.emboj.7600034 PMC1271672

[ref17] Ikai K., Takesako K., Shiomi K., Moriguchi M., Umeda Y., Yamamoto J., Kato I. and Naganawa H. (1991). Structure of Aureobasidin A. Journal of Antibiotics *(*Tokyo*)* 44, 925–933. 10.7164/antibiotics.44.925 1938614

[ref18] Kim S. K., Fouts A. E. and Boothroyd J. C. (2007). *Toxoplasma gondii* dysregulates IFN-gamma-inducible gene expression in human fibroblasts: insights from a genome-wide transcriptional profiling. Journal of Immunology 178, 5154–5165. 10.4049/jimmunol.178.8.5154 17404298

[ref19] Lauer S. A., Ghori N. and Haldar K. (1995). Sphingolipid synthesis as a target for chemotherapy against malaria parasites. Proceeds of the National Academy of Sciences USA 92, 9181–9185. 10.1073/pnas.92.20.9181 PMC40948 7568097

[ref20] Meissner M., Schluter D. and Soldati D. (2002). Role of *Toxoplasma gondii* myosin A in powering parasite gliding and host cell invasion. Science 298, 837–840. 12399593 10.1126/science.1074553

[ref21] Mina J. G., Pan S. Y., Wansadhipathi N. K., Bruce C. R., Shams-Eldin H., Schwarz R. T., Steel P. G. and Denny P. W. (2009). The *Trypanosoma brucei* sphingolipid synthase, an essential enzyme and drug target. Molecular and Biochemical Parasitology 168, 16–23. 19545591 10.1016/j.molbiopara.2009.06.002

[ref22] Mina J. G., Mosely J. A., Ali H. Z., Shams-Eldin H., Schwarz R. T., Steel P. G. and Denny P. W. (2010). A plate-based assay system for analyses and screening of the *Leishmania major* inositol phosphorylceramide synthase. International Journal of Biochemistry and Cell Biology 42, 1553–1561. 20561598 10.1016/j.biocel.2010.06.008

[ref23] Mina J. G., Mosely J. A., Ali H. Z., Denny P. W. and Steel P. G. (2011). Exploring Leishmania major inositol phosphorylceramide synthase (*Lmj*IPCS): insights into the ceramide binding domain. Organic and Biomolecular Chemistry 9, 1823–1830. 21267500 10.1039/c0ob00871k

[ref24] Nagiec M. M., Nagiec E. E., Baltisberger J. A., Wells G. B., Lester R. L. and Dickson R. C. (1997). Sphingolipid synthesis as a target for antifungal drugs. Complementation of the inositol phosphorylceramide synthase defect in a mutant strain of *Saccharomyces cerevisiae* by the AUR1 gene. Journal of Biological Chemistry 272, 9809–9817. 9092515 10.1074/jbc.272.15.9809

[ref25] Pratt S., Wansadhipathi-Kannangara N. K., Bruce C. R., Mina J. G., Shams-Eldin H., Casas J., Hanada K., Schwarz R. T., Sonda S. and Denny P. W. (2013). Sphingolipid synthesis and scavenging in the intracellular apicomplexan parasite, *Toxoplasma gondii*. Molecular and Biochemical Parasitology 187, 43–51. 23246819 10.1016/j.molbiopara.2012.11.007 PMC3629565

[ref26] Romano J. D., Sonda S., Bergbower E., Smith M. E. and Coppens I. (2013). *Toxoplasma gondii* salvages sphingolipids from the host Golgi through the rerouting of selected Rab vesicles to the parasitophorous vacuole. Molecular Biology of the Cell 24, 1974–1995. 23615442 10.1091/mbc.E12-11-0827 PMC3681701

[ref27] Salto M. L., Bertello L. E., Vieira M., Docampo R., Moreno S. N. and de Lederkremer R. M. (2003). Formation and remodeling of inositolphosphoceramide during differentiation of *Trypanosoma cruzi* from trypomastigote to amastigote. Eukaryotic Cell 2, 756–768. 12912895 10.1128/EC.2.4.756-768.2003 PMC178363

[ref28] Simons K. and Ikonen E. (1997). Functional rafts in cell membranes. Nature 387, 569–572. 9177342 10.1038/42408

[ref29] Soete M., Camus D. and Dubremetz J. F. (1994). Experimental induction of bradyzoite-specific antigen expression and cyst formation by the RH strain of *Toxoplasma gondii in vitro*. Experimental Parasitology 78, 361–370. 8206135 10.1006/expr.1994.1039

[ref30] Sonda S., Sala G., Ghidoni R., Hemphill A. and Pieters J. (2005). Inhibitory effect of Aureobasidin A on *Toxoplasma gondii*. Antimicrobial Agents and Chemotherapy 49, 1794–1801. 15855498 10.1128/AAC.49.5.1794-1801.2005 PMC1087623

[ref31] Takesako K., Ikai K., Haruna F., Endo M., Shimanaka K., Sono E., Nakamura T., Kato I. and Yamaguchi H. (1991). Aureobasidins, new antifungal antibiotics. Taxonomy, fermentation, isolation, and properties. Journal of Antibiotics *(*Tokyo*)* 44, 919–924. 10.7164/antibiotics.44.919 1938613

[ref32] Vacaru A. M., van den Dikkenberg J., Ternes P. and Holthuis J. C. (2013). Ceramide phosphoethanolamine biosynthesis in *Drosophila* is mediated by a unique ethanolamine phosphotransferase in the Golgi lumen. Journal of Biological Chemistry 288, 11520–11530. 23449981 10.1074/jbc.M113.460972 PMC3630839

[ref33] Webster J. P., Kaushik M., Bristow G. C. and McConkey G. A. (2013). *Toxoplasma gondii* infection, from predation to schizophrenia: can animal behaviour help us understand human behaviour? Journal of Experimental Biology 216, 99–112. 23225872 10.1242/jeb.074716 PMC3515034

[ref34] Welti R., Mui E., Sparks A., Wernimont S., Isaac G., Kirisits M., Roth M., Roberts C. W., Botte C., Marechal E. and McLeod R. (2007). Lipidomic analysis of *Toxoplasma gondii* reveals unusual polar lipids. Biochemistry 46, 13882–13890. 17988103 10.1021/bi7011993 PMC2576749

[ref35] Wuts P. G., Simons L. J., Metzger B. P., Sterling R. C., Slightom J. L. and Elhammer A. P. (2015). Generation of broad-spectrum antifungal drug candidates from the natural product compound Aureobasidin A. ACS Medical Chemistry Letters 6, 645–649. 10.1021/acsmedchemlett.5b00029 PMC4468416 26101567

[ref36] Young S. A., Mina J. G., Denny P. W. and Smith T. K. (2012). Sphingolipid and ceramide homeostasis: potential therapeutic targets. Biochemistry Research International 2012, 248135. 22400113 10.1155/2012/248135 PMC3286894

