# The antifungal Aureobasidin A and an analogue are active against the protozoan parasite *Toxoplasma gondii* but do not inhibit sphingolipid biosynthesis – Corrigendum

**DOI:** 10.1017/S0031182017000877

**Published:** 2018-02

**Authors:** A. Q. I. ALQAISI, A. J. MBEKEANI, M. BASSAS LLORENS, A. P. ELHAMMER, P. W. DENNY

The authors apologise for errors which appear in the legend of Figure 2. Please find below the corrected legend.

Fig. 2. ED50 of Aureobasidin A (AbA, A-D) or Compound 20 (Cpmd 20, E-H) – µg mL−1; (95% Confidence Interval) – against the *Toxoplasma* RH tachyzoite form in HFF cells. 6 days post addition of the compounds. In agreement with Sonda et al. (2005), both compounds were non-toxic to HHF cells under the conditions employed. A and E: no wash out post-compound addition; B and F: wash out 2 h post-compound addition; C and G: wash out 8 h post-compound addition; D and H: 2 h pre-treatment of isolated parasites pre-infection. Calculated using GraphPad Prism 7, log (inhibitor) vs normalized response – Variable slope. >10 µg mL−1 – ED50 could not be determined. Representative in triplicate dataset.
