# Supplementary material for: The antifungal Aureobasidin A and an analogue are active against the protozoan parasite Toxoplasma gondii but do not inhibit sphingolipid biosynthesis
Source: Parasitology. 2017 May 10;145(2):148–55. doi: 10.1017/S0031182017000506 (PMC5964465; doi:10.1017/S0031182017000506)

FIGURE S1

Quantitation of host (CHO) cell sphingolipid response to *Toxoplasma* challenge using real time PCR. No significant response in terms of the expression of key sphingolipid biosynthetic enzymes was detected.
LCB2 – subunit 2 of serine palmitoyltransferase (first and rate limiting step in sphingolipid biosynthesis); SMS 1 and 2 – sphingomyelin synthase 1 and 2. GoI – gene of interest; HkG – housekeeping gene. Replicate in triplicate data set.

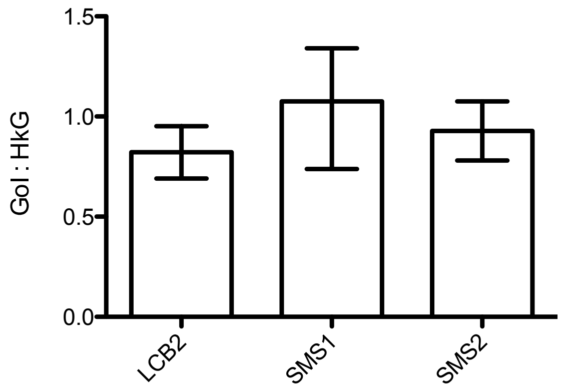

Supplement: Supplementary file 1 [file S0031182017000506sup001.pdf]
